# GMP-Compliant Universal Antigen Presenting Cells (uAPC) Promote the Metabolic Fitness and Antitumor Activity of Armored Cord Blood CAR-NK Cells

**DOI:** 10.3389/fimmu.2021.626098

**Published:** 2021-02-26

**Authors:** Enli Liu, Sonny O. T. Ang, Lucila Kerbauy, Rafet Basar, Indreshpal Kaur, Mecit Kaplan, Li Li, Yijiu Tong, May Daher, Emily L. Ensley, Nadima Uprety, Ana Karen Nunez Cortes, Ryan Z. Yang, Ye Li, Hila Shaim, Francia Reyes Silva, Paul Lin, Vakul Mohanty, Sunil Acharya, Mayra Shanley, Luis Muniz-Feliciano, Pinaki P. Banerjee, Ken Chen, Richard E. Champlin, Elizabeth J. Shpall, Katayoun Rezvani

**Affiliations:** ^1^Department of Stem Cell Transplantation and Cellular Therapy, The University of Texas MD Anderson Cancer Center, Houston, TX, United States; ^2^Departments of Stem Cell Transplantation and Hemotherapy/Cellular Therapy, Hospital Israelita Albert Einstein, Saõ Paulo, Brazil; ^3^Department of Genetics and Evolutionary Biology, Human Genome and Stem Cell Research Center, Biosciences Institute, University of Saõ Paulo, Saõ Paulo, Brazil; ^4^Department of Bioinformatics and Computational Biology, The University of Texas MD Anderson Cancer Center, Houston, TX, United States

**Keywords:** universal antigen presenting cell, cell engineering, K562 cells, adoptive cancer immunotherapy, NK cell expansion

## Abstract

Natural killer (NK) cells are innate lymphocytes recognized for their important role against tumor cells. NK cells expressing chimeric antigen receptors (CARs) have enhanced effector function against various type of cancer and are attractive contenders for the next generation of cancer immunotherapies. However, a number of factors have hindered the application of NK cells for cellular therapy, including their poor *in vitro* growth kinetics and relatively low starting percentages within the mononuclear cell fraction of peripheral blood or cord blood (CB). To overcome these limitations, we genetically-engineered human leukocyte antigen (HLA)-A^−^ and HLA-B^−^ K562 cells to enforce the expression of CD48, 4-1BBL, and membrane-bound IL-21 (mbIL21), creating a universal antigen presenting cell (uAPC) capable of stimulating their cognate receptors on NK cells. We have shown that uAPC can drive the expansion of both non-transduced (NT) and CAR-transduced CB derived NK cells by >900-fold in 2 weeks of co-culture with excellent purity (>99.9%) and without indications of senescence/exhaustion. We confirmed that uAPC-expanded research- and clinical-grade NT and CAR-transduced NK cells have higher metabolic fitness and display enhanced effector function against tumor targets compared to the corresponding cell fractions cultured without uAPCs. This novel approach allowed the expansion of highly pure GMP-grade CAR NK cells at optimal cell numbers to be used for adoptive CAR NK cell-based cancer immunotherapy.

**Graphical Abstract:**
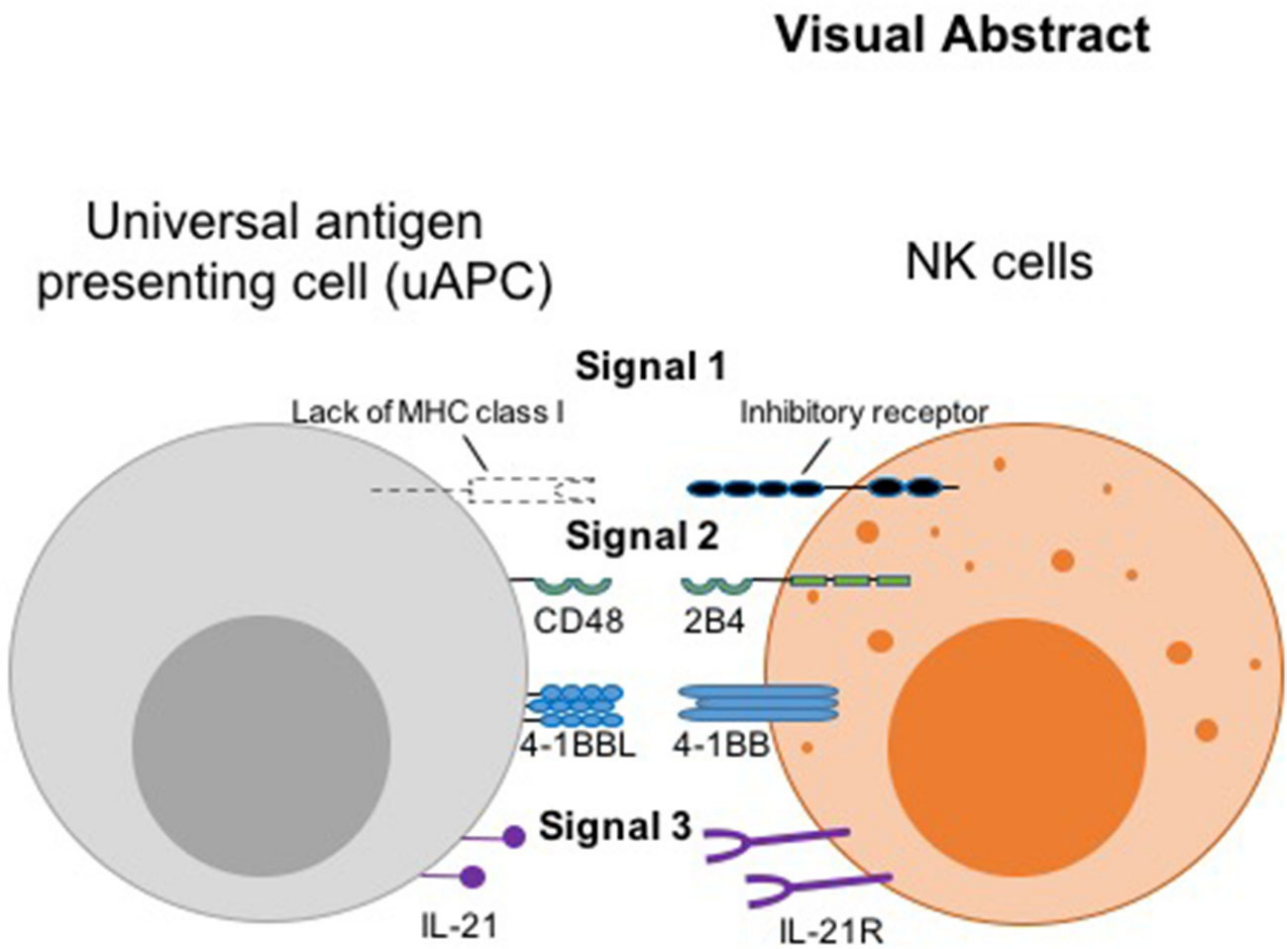


## Introduction

Remarkable advances have been made in the field of cellular therapy in recent years, including the Food and Drug Administration (FDA) approval of chimeric antigen receptor (CAR) T cell therapies as standard of care for the treatment of relapsed or refractory CD19^+^ malignancies including B cell acute lymphoblastic leukemia (B-ALL) (1), diffuse large B-cell lymphoma (DLBCL) (2), and mantle cell lymphoma (3). Efforts to replicate the success of anti-CD19 CAR-T cell therapies to other cancer types, including solid tumors, are ongoing. Challenges include the immunosuppressive tumor microenvironment as well as technical difficulties with manufacturing cells for immunotherapy such as histocompatibility mismatch and scale-up from research to clinical-grade immune cell production. Like T cells, natural killer (NK) cells are immune effector cells with potent anti-tumor capabilities. Unlike T cells, however, NK cells do not produce graft-vs.-host disease (GvHD) and their use has not been associated with serious toxicities, such as cytokine release syndrome (CRS) or immune-effector cell-associated neurotoxicity syndrome (ICANS) (4, 5). NK cell-based immunotherapy has been tested in an array of human diseases ranging from non-malignant viral and non-viral infections, to malignant indications including lymphoma (1), leukemia (6, 7), myeloma (1), carcinoma (2, 6–8), and sarcoma (9, 10). While NK cells hold promise for the treatment of cancer, the ever-expanding plethora of activating and inhibitory receptors with overlapping functions complicates our understanding of NK cell triggering and activation (Supplementary Figure 1). While NK cell signaling is more complicated than the conventional three-signal model of T cell activation, the similarities, such as the well-characterized interaction nexus between effector NK cells and endogenous antigen presenting cells, help delineate the minimal requirements for conditioning, priming, expanding, and triggering NK cells to kill.

Thus, we hypothesized that the initiating signal postulated by the missing-self hypothesis (8) can serve as a universal surrogate “signal 1,” while the co-activating receptors, 2B4 and 4-1BB (11, 12) can serve as “signal 2.” We designated IL-21R (13) as receiver for “signal 3,” the cytokine stimulus, which complements another important cytokine, IL-15, genetically engineered for constitutive secretion in our CAR NK cells (9) (Visual Abstract). Thus, we engineered CD48, 4-1BB ligand, and membrane-bound IL-21 (mbIL-21) onto a K562-based presenter cell to serve as counter ligands for the three signaling receptors. This minimal set of antigenic stimulators forms our universal feeder cells for robust expansion of both research and clinical grade non-transduced and armored CAR NK cells, irrespective of the antigen the CAR molecule is targeting.

## Materials and Methods

### Cell Lines

K562, an erythroleukemia cell line, (10) was obtained from the University of Tennessee (10) and the identity was validated by STR DNA fingerprinting using the AmpFLSTR Identifiler kit according to manufacturer instructions (Applied Biosystems Catalog# 4322288). The STR profiles were compared to fingerprint data on the ATCC fingerprint database (ATCC.org) and the German Collection of Microorganisms and Cell Cultures GmbH (DSMZ, Germany). Raji, a Burkitt lymphoma cell line (14), was purchased from American Type Culture Collection (Manassas, VA, USA). K562 and Raji cells were cultured in Roswell Park Memorial Institute (RPMI) medium supplemented with 10% fetal bovine serum (FBS), 1% penicillin-streptomycin and 2 mM L-glutamine.

### Retroviral Transfer Construct Synthesis

The retroviral vectors encoding CD48, CD137-ligand (4-1BBL), and membrane-bound interleukin (IL)-21 (mbIL-21) were synthesized to specifications (GeneArt, Germany). Transient retroviral supernatants were produced as previously described (1). K562 cells were serially transduced with retroviral supernatants harboring CD48, CD137-ligand, and mbIL-21 transgenes and limiting-dilution cloning performed after each transduction to isolate specific clones for further characterizations. The pSFG retroviral vector encoding anti-CD19 CAR in combination with the human *IL15* gene and the inducible caspase-9 suicide gene separated using 2A sequence peptides (iC9.CAR19.CD28-zeta-2A-IL-15) has been previously described (6, 7) and was kindly provided by Dr. Gianpietro Dotti (University of North Carolina).

### Flow Cytometry

Cells were incubated with designated antibodies for 20 min at 4°C, washed, and resuspended in staining buffer before data acquisition using a LSR II/Fortessa cytometer (BD Biosciences, San Jose, CA), and analyzed using FlowJo software (BD Life Sciences, USA). uAPC were stained with antibodies against IL-21, CD48, and 4-1BB ligand (see Supplementary Table 1 for details of antibodies) to detect and quantify the enforced transgene expression.

### NK Cell Isolation

Cord blood (CB) units for research were obtained by the MD Anderson Cancer Center CB Bank, under protocols approved by the institutional review board. Healthy human peripheral blood units were sourced from Gulf Coast Regional Blood Center (Houston, TX). CB and peripheral blood mononuclear cells (PBMCs) were isolated by a density-gradient centrifugation (Ficoll-Histopaque; Sigma, St Louis, MO, USA). CD56-positive NK cells were purified using an NK isolation kit (Miltenyi Biotec, Inc., San Diego, CA, USA), and were stimulated with irradiated (100 Gy) uAPCs (feeder cell to NK cell ratio of 2:1) and recombinant human IL-2 (Proleukin, 200 U/ml; Chiron, Emeryville, CA, USA) in complete serum-free stem cell growth medium (SCGM) (CellGenix GmbH, Freiburg, Germany) on day 0. In specific comparative experiments, uAPC was substituted with C9/IL-21 (15), a previously characterized feeder cell also harboring mbIL21. Activated NK cells were transduced with retroviral supernatants on day +6 in human fibronectin-coated plates (Clontech Laboratories, Inc., Mountain View, CA, USA). Three days later (on day +9), NK cells were stimulated again with irradiated uAPC and IL-2. On day +15, CAR-transduced NK cells were collected for use in the indicated assays.

### NK Cell Phenotyping and Functional Assay

NK cell growth was evaluated over 14–21 days of *ex vivo* culture and counted using trypan blue exclusion for viability every 3 days. NK cells were assessed for expression of CD48, IL-21, CD137L (4-1BBL), CD14, CD45 and CD32 (to identify K562) by FACS (see Supplementary Table 1 for details of antibodies). CAR-transduced CB-NK cells were stained with Alexa-Fluor647 affinity-purified F (ab')2 fragment goat anti-human IgG (H+L) antibody (CAR Ab) (Jackson ImmunoResearch, West Grove, PA, USA) for CAR expression. In addition, intracellular cytokines were also measured on day 14 of culture using *ex vivo*-expanded non-transduced (NT) and iC9/CAR.19/IL-15 (CAR) NK cells. To assess effector function, NK cells were co-cultured at 0.25 × 10^6^ cells/well for 5 h in 96-well plates with Raji cells or K562 targets (positive control) at an effector to tumor cell ratio (E:T ratio) of 5:1. CD107a degranulation and intracellular cytokine production were measured as previously described (11).

### NK Cell Cytotoxicity Assay

To assess cytotoxicity, *ex vivo*-expanded research- or Good Manufacturing Practice (GMP)-grade NT and iC9/CAR.19/IL-15 (CAR) transduced NK cells were co-cultured with ^51^Cr-labeled Raji and K562 targets (positive control) at different E:T ratios. The cytotoxicity was measured by ^51^Cr release as previously described (12).

### Mouse Xenograft Model

We used a NOD/SCID IL-2Rγ null (NSG) xenograft model (9), with the aggressive NK-resistant Raji cell line, to assess the *in vivo* anti-tumor effect of CAR-transduced CB-NK cells. Adult NSG mice (10–12 weeks old; Jackson Laboratories, ME) were γ-irradiated with 300 cGy and inoculated intravenously (i.v.) on day 0 with Raji cells (2 × 10^4^) stably-transduced with firefly luciferase (ffluc) transgene. Freshly expanded CAR-transduced CB-NK cells, using either uAPC or C9/IL-21 as feeders, were then injected through the tail vein at a dose of 3 × 10^6^ cells in 200 μL volume on days 0. Mice were subjected to weekly bioluminescence imaging (BLI; Xenogen-IVIS 200 Imaging system; Caliper, Waltham, MA), to access the status of engrafted tumor persistence. All mouse experiments were performed in accordance with protocols approved by the Institutional Animal Care and Use Committee.

### RNA Sequencing (RNA-Seq)

RNA was extracted and purified (RNeasy Plus Mini Kit, Qiagen) from 5 × 10^6^ *ex vivo*-expanded NT and iC9/CAR.19/IL-15 (CAR) purified NK cells at day 0 (baseline) or 15 days after expansion with uAPC. SmartSeq2 RNA-seq libraries were prepared as described (13). The indexed libraries were pooled and sequenced with 50-bp paired-end reads using Illumina HiSeq 2500 (16).

RNA-seq counts were used to perform differential expression analysis using DESeq2 (v1.22.2), including Gene Set Enrichment Analysis (GSEA), to look for differences in biological states of NK cells before and after uAPC stimulations using a pre-defined set of genes. The differential expression analysis algorithm used controls for sample specific variability while comparing expression between two experimental conditions. Differentially expressed genes were identified at a *P* < 0.01 and absolute log_2_-fold change >2, normalized counts for these genes was z-transformed and plotted as a heatmap using the pheatmap package (v 1.0.12). GSEA was carried out using gage (v 2.32.1) and the stat column from the DESeq result was used as the input, differentially expressed pathways were identified at *P* < 0.01. In bar plot, a positive “stat mean” indicates upregulation and a negative “stat mean,” down-regulation. The gene level boxplots are of normalized counts and was plotted with ggplot2 (v 3.2.1).

### Mass Cytometry

Antibodies used for mass cytometry analysis are listed in Supplementary Table 2. Antibodies sourcing, labeling, and cell staining were performed as described previously (17). Briefly, antibodies were labeled with metal-tag at the MD Anderson Cancer Center Flow and Mass Cytometry Core Facility using the MaxPar Antibody Labeling kit from Fluidigm (catalog #201300). Mass cytometry data were normalized based on EQTM four element signal shift over time using Fluidigm normalization software 2. Initial data quality control was performed using Flowjo version 10.2. Calibration beads were gated out and singlets were chosen based on iridium 193 staining and event length. Dead cells were excluded by the Pt195 channel and further gating was performed to select CD45^+^ cells and then the NK cell population of interest (CD3^−^CD56^+^). t-SNE analysis was performed using automated dimension reduction, including (viSNE) in combination with FlowSOM for clustering of resting NK cells (unstimulated, day 0) or following co-culture with uAPCs for 14 days (Expanded NK cells).

### uAPC Validation for Regulatory Compliance and Use in GMP

Validation plans and procedures were prepared in accordance with FDA guidance (18, 19) to comply with the Chemistry, Manufacturing, and Control (CMC) Information requirements for Human Gene Therapy Investigational New Drug Applications (INDs), and to control for critical processes, installations, operational and performance qualifications governing cell products (20). Both universal and product specific characterization was maintained, as well as phase-specific requirements. In-process and end product testing included: sterility, endotoxin, mycoplasma (PCR testing at the time of cell harvesting), viability, identity (to identify specific cell types by flow cytometry), and *in vitro* functional killing.

### GMP NK Cell Expansion With uAPC

NK cells were expanded using the G-Rex® bioreactor (Saint Paul, Minnesota, USA) in co-cultures with uAPC for the CB-NK CAR clinical trial (21) (NCT Number: NCT03056339). This closed cell culture system enables using flasks with a gas-permeable membrane, allowing for optimal gaseous exchange and ensuring aerobic growth kinetics. Using previously optimized variables which included cell seeding density, calibrated media volume, and media formulation, we were able to adapt the G-Rex M100 series from pre-clinical protocols to translate and linearly scale the procedure to the desired yield in the GMP setting.

### Telomere Length Analysis

The telomere length in CB-NK cells was assayed by quantitative PCR using the Absolute Human Telomere Length Quantification qPCR Assay Kit (ScienCell Research Laboratories, Carlsbad, CA 92008, Catalog# 8918) and following the manufacturer's instructions.

### Statistical Analyses

Standard error of the mean (SEM) was used to compare differences in CB NK cell fold expansion and effector function. Statistical significance is determined as *p* ≤ 0.05.

## Results

### Generation and Optimization of K562-Based Feeder Cells Retrovirally Transduced to Co-express 4-1BBL, CD48, and Membrane Bound IL-21

**Figure 1:**
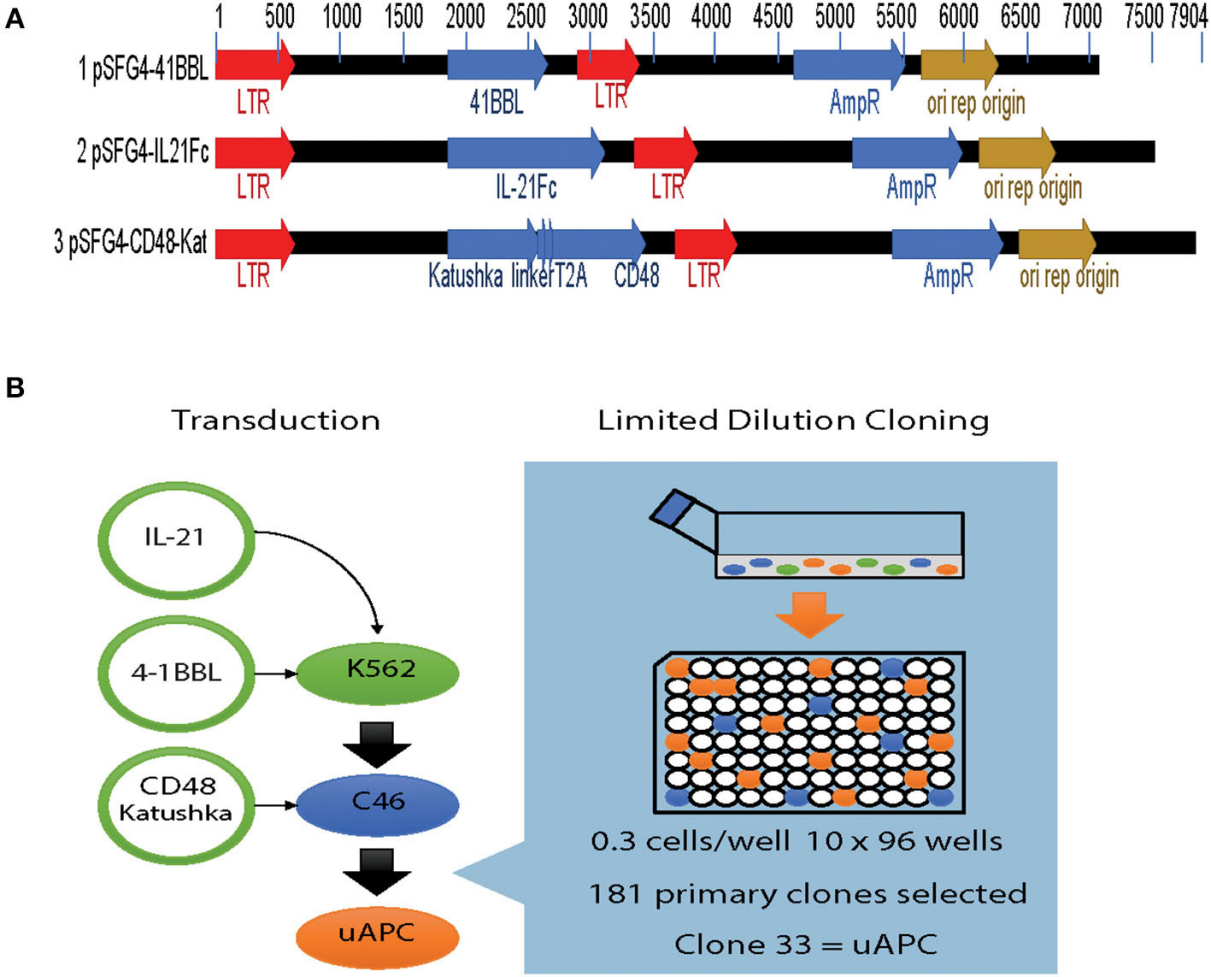
Generation of the K562-based universal antigen presenting cell (uAPC). **(A)** Retroviral transfer vectors used to introduce the transgenes IL-21, 4-1BBL (CD137L), CD48, and Katushka to enforce their expression in K562. **(B)** Experimental schema summarizing our strategy to generate uAPC. Transgenes were retrovirally transduced serially into K562 cells, and limiting dilution cloning performed after each transduction to derive uAPC. Clone 46 (C46) is an intermediate clone positive for membrane-bound IL-21 and 4-1BBL. uAPC is positive for membrane-bound IL-21, 4-1BBL, and CD48.

Parental K562 cells were transduced with retroviral vectors expressing membrane bound interleukin 21 (mbIL-21), 4-1BBL, and CD48 transgenes individually (Figure 1A). Briefly, viral supernatants were prepared and used to sequentially transduce K562 cells. After each stable transduction, limiting dilution cloning was performed and the best characterized clone was used for the next round of manipulation (Figure 1B). By serial transductions and repeated cloning, each individual transgene integrates independently into specific genomic loci, ensuring no interference in expression from other transgenes. After introduction of mbIL-21 followed by 4-1BBL, we grew out a suitably stable intermediate cell line termed clone 46 or C46 (Figure 1B). This clone was selected by limiting dilution and tested functionally for optimal levels of transgenes expression, as well as to provide for optimal induction of NK cell proliferation. A final round of transduction with the CD48 construct, which also carries a fluorescent marker (Katushka), yielded a triple-positive cell population ready for further characterization (Figure 1B). These triple-positive cells were immunophenotyped by flow cytometry, followed by another round of cell cloning which yield 181 primary clones with clone 33 selected as the most suitable for further characterization, which we subsequently termed the universal antigen presenting cell or uAPC (Figure 1B).

**Figure 2:**
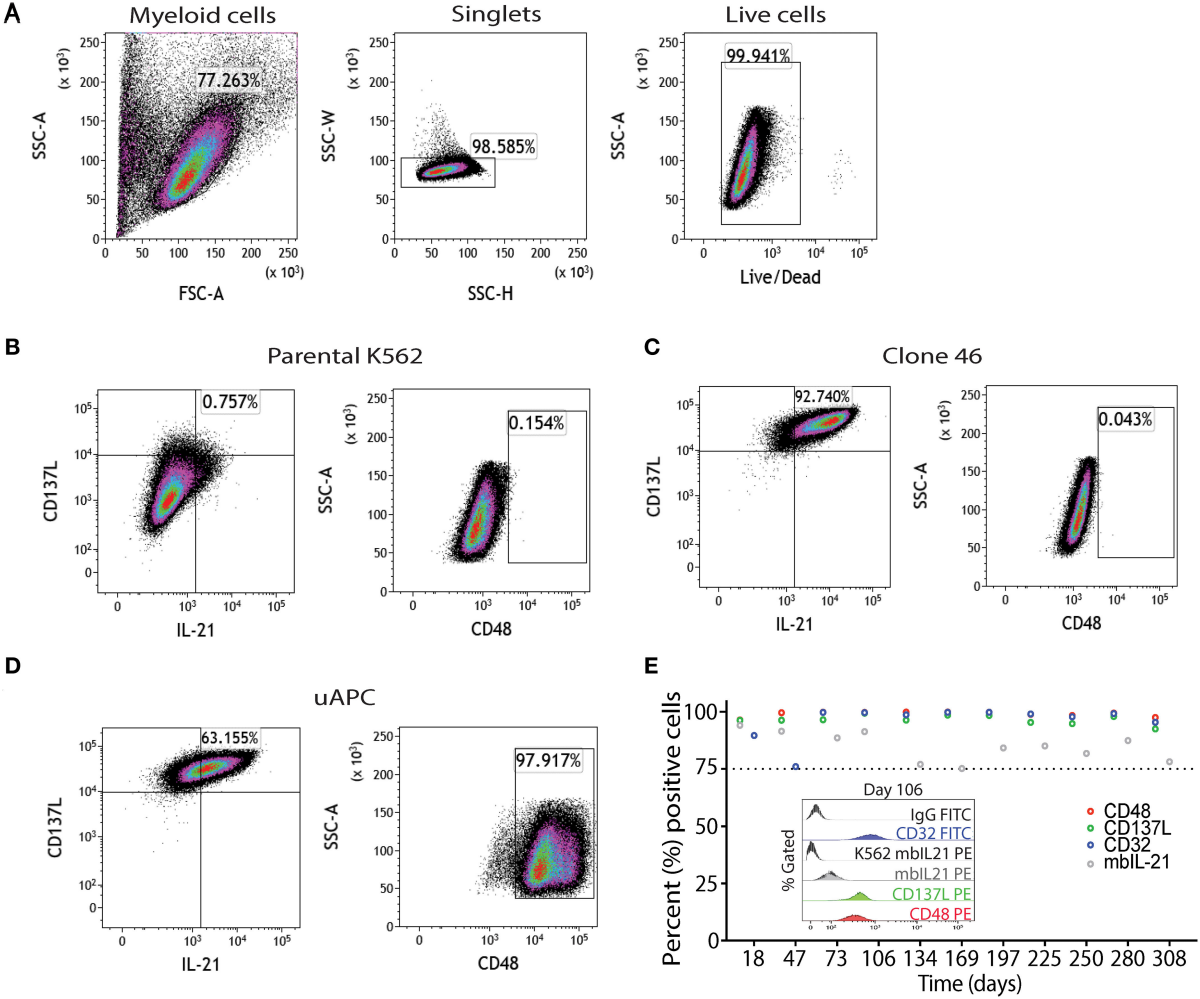
Phenotypic characterization of uAPC. **(A)** Representative fluorescence-activated cell sorting (FACS) plots showing gating strategy to identify uAPC using FlowJo software (V10.5). Briefly, forward scatter and side scatter plot was used to gate on myeloid cells; next doublets were excluded using SSC-W/SSC-H and live cells were selected using Live/Dead dye. Representative FACS plots of the parental K562 cells (**B**, negative for all transgenes), clone 46 (**C**, IL-21^+^CD137L^+^), and uAPC (**D**, IL-21^+^CD137L^+^CD48^+^Katushka^+^ cells). **(E)** The expression levels of CD48 (red circles), CD137L (green circles), membrane-bound IL-21 (mbIL-21; gray circles) and CD32 (blue circles) were assessed periodically by flow cytometry from aliquots of continuously cultured uAPC to determine stability of integrated transgenes. Inset histogram depicts the expression of indicated markers at day 106.

Parental K562, clone 46, and uAPC cells were immunophenotyped using a flow cytometry gating strategy as outlined in Figure 2A. Briefly, “myeloid”-gated cells were selected for single cells and the live cells were studied for expression of mbIL-21, 4-1BBL (CD137L), and CD48 (Figures 2B–D). The mbIL-21, 4-1BBL, and CD48 transduction efficiency in uAPC was >75% and remained stable for over 300 days (Figure 2E). CD32 was used to confirm the identity of the K562 cells. Thus, these data confirm the generation of a K562-based uAPC cell line stably co-expressing mbIL-21, 4-1BBL, and CD48. We also confirmed that the doubling time of our genetically engineered K562-based uAPC ranges from 23.26 to 26.42 h (*n* = 3 independent experiments) which is consistent with previous reports for parental K562 and derivative cell lines (10).

### uAPC Enhances the Antitumor Activity of Research-Grade Armored CAR-NK Cells

**Figure 3:**
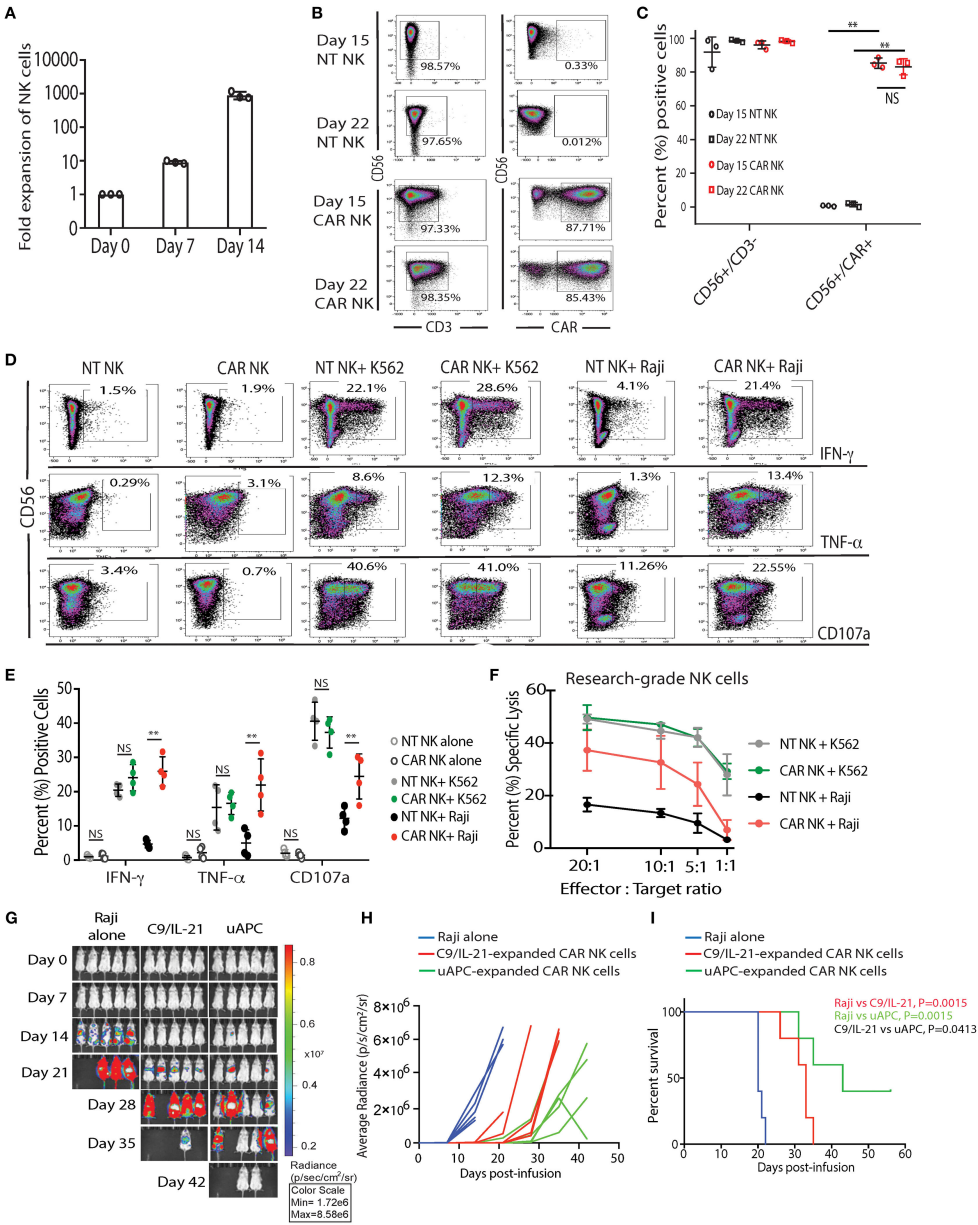
uAPC promotes the proliferation and cytotoxicity of research-grade NT and iC9/CAR19/IL-15 NK cells. **(A)** Fold expansion of research grade CB NK cells at day 0 (baseline) or after co-culture with uAPC for 7 or 14 days. Bars represent standard error of the mean (SEM) (*n* = 3 independent experiments). **(B)** Representative FACS plots showing the expression of research grade NT NK cells (CD56^+^CD3^−^) or iC9/CAR.19/IL-15 NK cells (CAR NK; CD56^+^CD3^−^CAR^+^) at days 15 and 22 of co-culture with uAPC. Inset numbers are the percentages (%) positive cells within the indicated regions. **(C)** Graph summarizing the data from **(B)**. Each circle or square represents an independent experiment (*n* = 3). Statistical significance is determined as **p ≤ 0.01. Bars represent standard error of the mean (SEM). NS, not significant. **(D)** Representative FACS plots of cytokine production (IFN-γ, TNF-α) and CD107a degranulation by uAPC-expanded (15 days) research grade NT or iC9/CAR19/IL-15 (CAR) NK cells followed by co-culture with or without K562 or Raji targets for 4 h (*n* = 4 independent experiments). Inset numbers in panels are the percentages of IFN-γ, TNF-α and CD107a-positive NK cells within indicated regions. **(E)** Graph summarizing the flow cytometry data on cytokine production (IFN-γ and TNF-α) and CD107a degranulation by uAPC-expanded research grade NT or iC9/CAR19/IL-15 (CAR) NK cells after co-culture with Raji target cells for 4 h. Each data point represents an independent experiment (*n* = 4). Statistical significance is indicated as **p ≤ 0.01. NS, not significant. Bars represent standard error of the mean (SEM). **(F)** Research grade NT or iC9/CAR.19/IL-15 (CAR) NK cells were expanded with uAPC for 15 days then co-cultured for 4 h with K562 or Raji targets at different effector: target ratios (E:T) and their cytotoxicity was determined by ^51^Cr release assay (*n* = 3). Error bars are determined by standard error of the mean (SEM). **(G)** iC9/CAR.19/IL-15 (CAR) NK cells were expanded with uAPC or C9/IL-21 for 15 days then injected into NSG mice engrafted with ffluc^+^-Raji tumor cells. Bioluminescence imaging was used to monitor the tumor growth of NSG mice treated with Raji alone, Raji plus C9/IL-21-expanded CAR NK cells or Raji plus uAPC-expanded CAR-NK cells. CAR-NK cells *ex vivo* expanded with uAPC resulted in superior anti-tumor control compared to animals receiving C9/IL-21-expanded CAR NK cells. Colors on linearly scaled bar indicate intensities of luminescence (red, highest; blue, lowest), correspond with intravital tumor burdens. The average radiance **(H)** and survival curves **(I)** are shown for the three groups of mice (*n* = 5 mice per group) and displayed for comparison. **(I)** Mice receiving uAPC-expanded CAR-NK cells had significantly longer survival compared to animals treated with CAR-NK cells expanded with C9/IL-21. The *p*-value in red represents the difference in survival between mice treated with C9/IL-21-expanded CAR NK cells compared to tumor control (*P* = 0.0015); the *p*-value in green represents the difference in survival between mice treated with uAPC-expanded CAR NK cells compared to tumor control (*P* = 0.0015); the *p*-value in black represents the difference in survival between mice treated with uAPC-expanded CAR NK cells compared to C9/IL-21-expanded CAR NK cells (*P* = 0.0413).

First, we tested whether uAPC can promote the *ex-vivo* expansion of CB-NK cells. We observed a 9-fold (median = 8.96, range = 8.26–10.1) and 903-fold expansion (median = 784, range = 752–1,174) of research grade NK cells after co-culture with uAPC for 7 and 14 days, respectively (Figure 3A). Importantly, both non-transduced (NT) controls and iC9/CAR.19/IL-15 (CAR-transduced) research grade NK cells co-cultured with uAPC exhibited high NK cell purity (>97%) at days 15 and 22 of co-culture with uAPC as determined by flow cytometry (Figures 3B,C). Moreover, uAPC-expanded iC9/CAR.19/IL-15 (CAR)-NK cells retained their CAR expression (>85%) (Figures 3B,C), produced significantly higher levels of IFN-γ and TNF-α and displayed greater degranulation (CD107a) and cytotoxicity against CD19^+^ Raji lymphoma cells compared to NT NK cells (Figures 3D–F). CAR NK cells were as efficient as NT NK cells in killing CD19-negative K562 targets (Figure 3F), indicating that the innate NK cell killing mechanisms remained unaltered after uAPC stimulation. We also tested the ability of uAPCs to support NK cell expansion beyond day 14. NK cells were expanded with uAPC + IL-2 for 1 week. The cells were then harvested and cultured long-term with IL-2 (100 U/ml) in the presence or absence of weekly uAPC feeder cells. As shown in Supplementary Figure 2, CAR-NK cells failed to expand with IL-2 alone and in the absence of weekly feeder cell support.

In addition, we compared the telomere length, *in vitro* and *in vivo* activity of CAR-transduced CB-NK cells expanded with uAPC with a previously reported K562-derived APC (C9/IL-21) expressing 4-1BBL and mbIL21 (15). We observe no significant differences in the telomere length (Supplementary Figure 3), fold expansion, or cytotoxicity of NK cells generated using the two feeder cell lines *in vitro* (Supplementary Figures 4, 5). Importantly, uAPC-expanded CAR-NK cells exerted superior antitumor activity *in vivo* in a Raji xenograft mouse model (Figures 3G–I). In a direct comparison, using CAR NK cells generated from the same CB donor, mice receiving CAR-NK cells expanded with uAPC achieved better tumor control (Figures 3G,H) and survived significantly longer (Figure 3I) when compared to animals treated with CAR-NK cells generated with C9/IL-21. Taken together, these data support the notion that a three-signal model of NK cell activation enables robust expansion of NK cells with greater antitumor potency.

### NK Cell Phenotypic and Molecular Signature Associated With uAPC Stimulation

**Figure 4:**
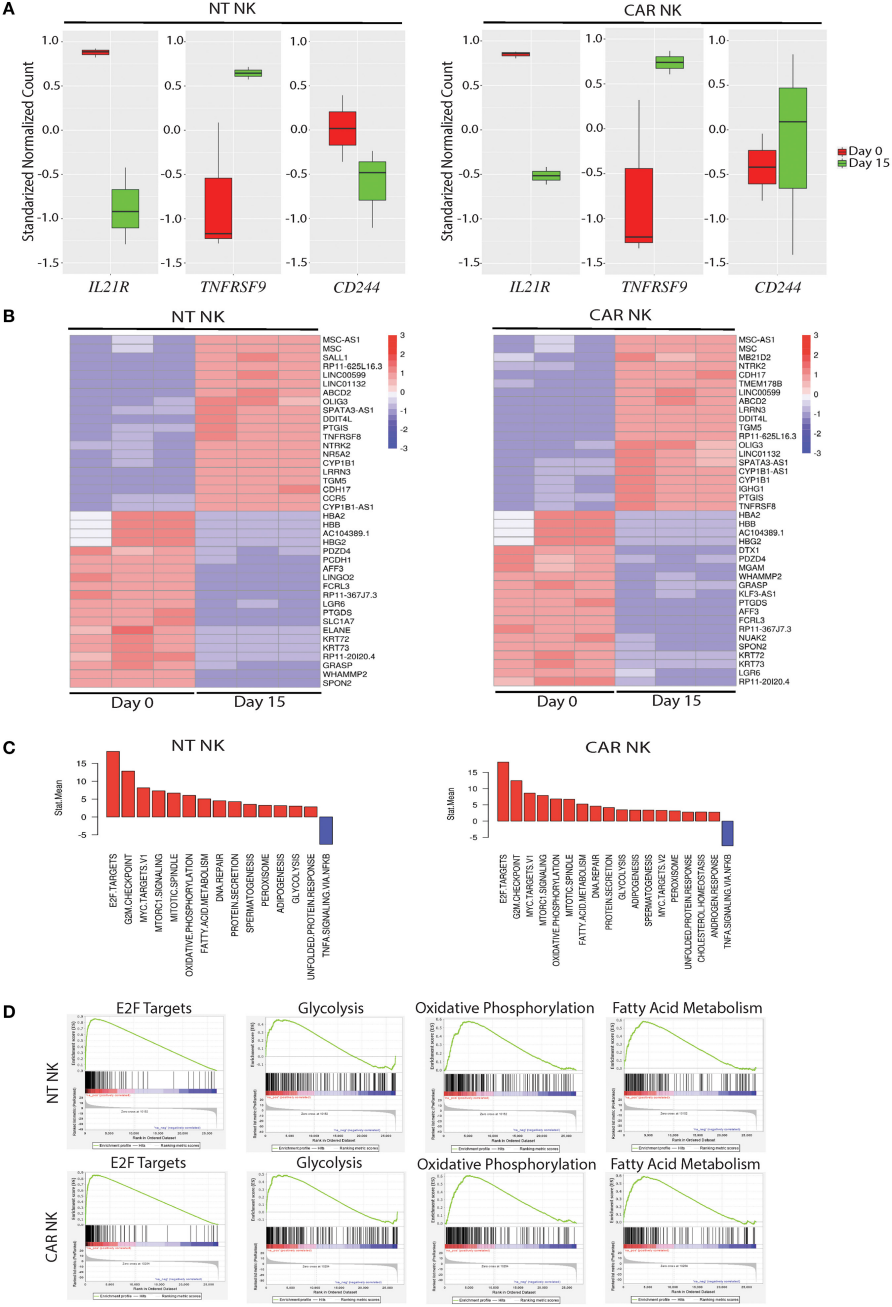
Molecular characterization of uAPC-expanded NT and iC9/CAR19/IL-15 NK cells. **(A)** RNA sequencing data showing standardized normalized counts of *Interleukin 21 Receptor* (*IL21R*), *TNF Receptor Superfamily Member 9* (*TNFRSF9*; *CD137; 4-1BB*) and *CD244 (2B4)* genes of NT (left) or iC9/CAR19/IL-15 (CAR) NK cells (right) at day 0 (unstimulated; red) and day 15 after stimulation with uAPC (green). **(B)** Heatmaps displaying RNA sequencing data of differentially expressed genes in purified NT (left) or iC9/CAR.19/IL-15 (CAR, right) NK cells at baseline (Day 0) or Day 15 after co-culture with uAPC (*n* = 3). Color scale reflects comparative RNA transcript levels, with red representing higher expression and blue representing lower expression. **(C)** Comparative Mean T Statistic bar graphs of RNA sequencing data showing the expression of pathways in NT (left) or iC9/CAR.19/IL-15 (CAR) NK cells (right) that are significantly increased (red) or significantly decreased (blue) upon stimulation with uAPC for 15 days. **(D)** Gene set enrichment analysis (GSEA) plots showing enrichment in E2F targets, glycolysis, oxidative phosphorylation and fatty acid metabolism in uAPC-activated NT NK cells (top plots) and iC9/CAR19/IL-15 (CAR) NK cells (bottom plots).

To gain insight into the transcriptomic and signaling pathways that accompany uAPC stimulation in NK cells, we performed RNA sequencing studies from purified NT and CAR NK cells at day 0 (baseline) and at day 15 after co-culture with uAPC. Stimulation with uAPC led to downregulation of the IL-21 receptor gene (*IL21R*) in both NT and CAR NK cells (Figure 4A), consistent with reports that IL-21 drives NK expansion (15) and modulates expression of NK cell receptors (22). On the other hand, *TNFRSF9* (4-1BB) was significantly induced (*P* < 0.05) in NT and CAR NK cells after stimulation with uAPC (Figure 4A) in keeping with its co-stimulatory role (23) and consistent with its importance in NK cell regulation. Lastly, 2B4 (*CD244*), a non-MHC binding counter receptor to CD48 was readily detectable both before and after exposure to uAPC (Figure 4A).

Transcriptomic profiling of both NT and CAR NK cells after exposure to uAPC (Day 15) revealed upregulation of genes related with effector function compared to baseline (Day 0). Importantly, we did not observe any pathway related to exhaustion, anergy, or senescence (Figure 4B). Genes related to cell cycling, cell membrane morphology and metabolic pathways were similarly increased in both uAPC-stimulated NT and CAR NK cells (Figure 4C). Gene-set enrichment analysis (GSEA) revealed an enrichment of genes involved in E2F targets, glycolysis, oxidative phosphorylation and fatty acid metabolism in uAPC-activated NT and CAR NK cells (Figure 4D). Of particular note, these metabolic mobilization patterns, in concert with lipid biogenesis and cell membrane reorganization, point to an active program indicative of cellular proliferation as well as priming of the effector components within NK cells to prepare for cytotoxic activities (24). Two-dimensional principal component analysis (PCA), with eigenvalue cutoffs of 1 and 2, can readily differentiate samples by their distinct states (Supplementary Figure 6), further supporting the non-clonal nature of our expanded NK cells.

**Figure 5:**
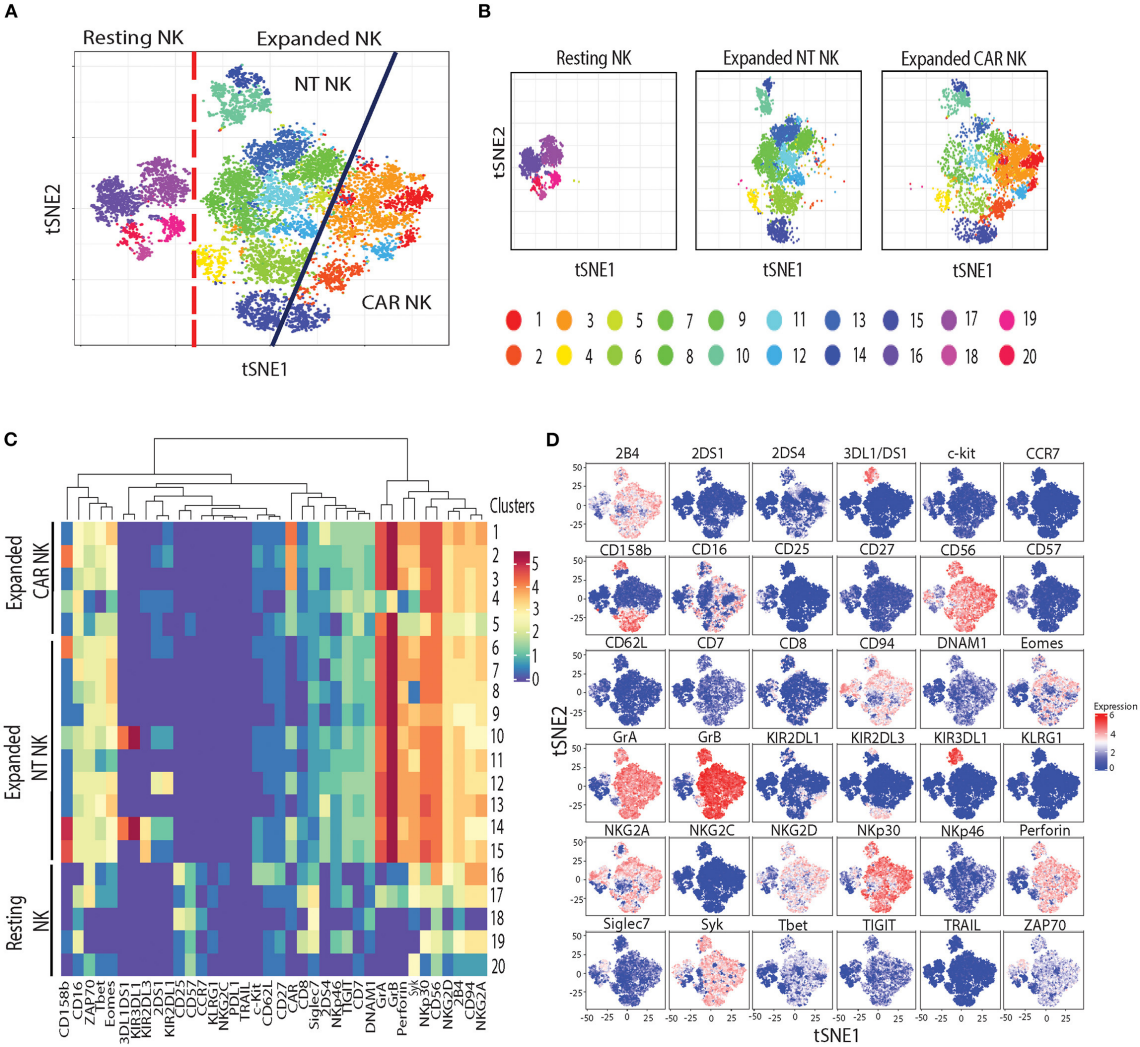
Phenotypic characterization of resting vs. uAPC-expanded NK cells. **(A)** The t-SNE map generated from FlowSOM analysis showing the 20 clusters of resting NK cells (freshly isolated at day 0) and uAPC-expanded NK cells (NT and iC9/CAR.19/IL-15 NK cells) for 14 days. **(B)** Individual t-SNE maps showing clusters corresponding to resting NK cells (left panel) or expanded NT NK cells (center panel) vs. expanded iC9/CAR19/IL-15 (CAR) NK cells (right panel) for 14 days with uAPC. **(C)** Comparative heatmap of mass cytometry data showing the expression of NK cell surface markers, cytotoxicity markers and transcription factors in resting NK cells and uAPC-expanded NK cells [NT and iC9/CAR19/IL-15 (CAR)]. Each column reflects the expression of a certain NK cell marker for each annotation and each row represents a separate cluster identified by FlowSOM analysis. Color scale shows the expression of each marker, with red representing higher expression and purple lower expression. **(D)** Individual t-SNE maps showing the expression of NK cell markers for resting NK cells vs. uAPC- expanded NK cells (NT and iC9/CAR19/IL-15 NK cells). Color scale indicates the signal intensity, ranging from low (blue) to high (red) after arcsine transformation.

Next, to assess the phenotypic changes associated with uAPC expansion in NK cells, we used cytometry by time-of-flight (CyToF) and a panel of 36 antibodies against inhibitory and activating receptors, as well as differentiation, homing and activation markers. We identified 20 distinct clusters of NK cells characterizing resting, uAPC-expanded NT and CAR NK cells (Figures 5A,B). Resting NK cells were primarily composed of clusters 16–20, whereas clusters 1–5 were exclusively observed in expanded CAR NK cells and clusters 6–15 were shared between expanded NT and CAR NK cells (Figures 5A,B). Both uAPC-expanded NT and CAR NK cells displayed increased expression of markers of activation and cytotoxicity, including granzyme A (GrA), granzyme B (GrB), perforin; transcription factors (Eomes, T-bet) and activating receptors (NKp30, NKG2D, 2B4, CD94/NKG2C) when compared to resting NK cells (Figures 5C,D). Together, these data suggest that culture with uAPC enhances the activation and anti-tumor activity of both NT and CAR NK cells while retaining their CAR specificity.

### Validation and Characterization of Clinical-Grade uAPC for Use in cGMP

In order to use uAPC in a cGMP setting for generation of NK cell-based therapeutic products, we performed a series of validation runs based on release criteria in accordance with current best practices. Our release criteria as listed in Table 1, including sterility, viability, identity and potency, were established and assayed as shown to meet regulatory requirements. In addition, a standard operating procedure to handle and use uAPC in facilitating NK expansion *ex vivo* for clinical production was established to aid in quality assurance and quality control. As part of our effort to ensure the integrity of uAPC in clinical use, we also genetically fingerprinted the uAPC and showed that the STR profiles matched that of the K562 parental line (Supplementary Table 3).

**Table 1:** List of validation tests to qualify uAPC for use in GMP facility.

**Test**	**Specification**
Sterility/bacteriology (cell product and supernatant) by BACTEC culture (aerobic and anaerobic)	Negative at 14 days
Endotoxin (cell product) by limulus amebocyte lysate (LAL) assay	<1.0 EU/ml
Mycoplasma (cell product and supernatant medium) by PCR	Negative
Viability (cell product) by dye (sytox™ green) exclusion	≥70% viable
Identity (immunophenotyping) by flow cytometry	≥75% Expressing CD137L^+^ ≥75% Expressing IL-21^+^ ≥75% Expressing CD48^+^ ≥75% Expressing CD32^+^
Potency (^51^Cr-release cytotoxicity assay)	Positive killing of target cells

**Figure 6:**
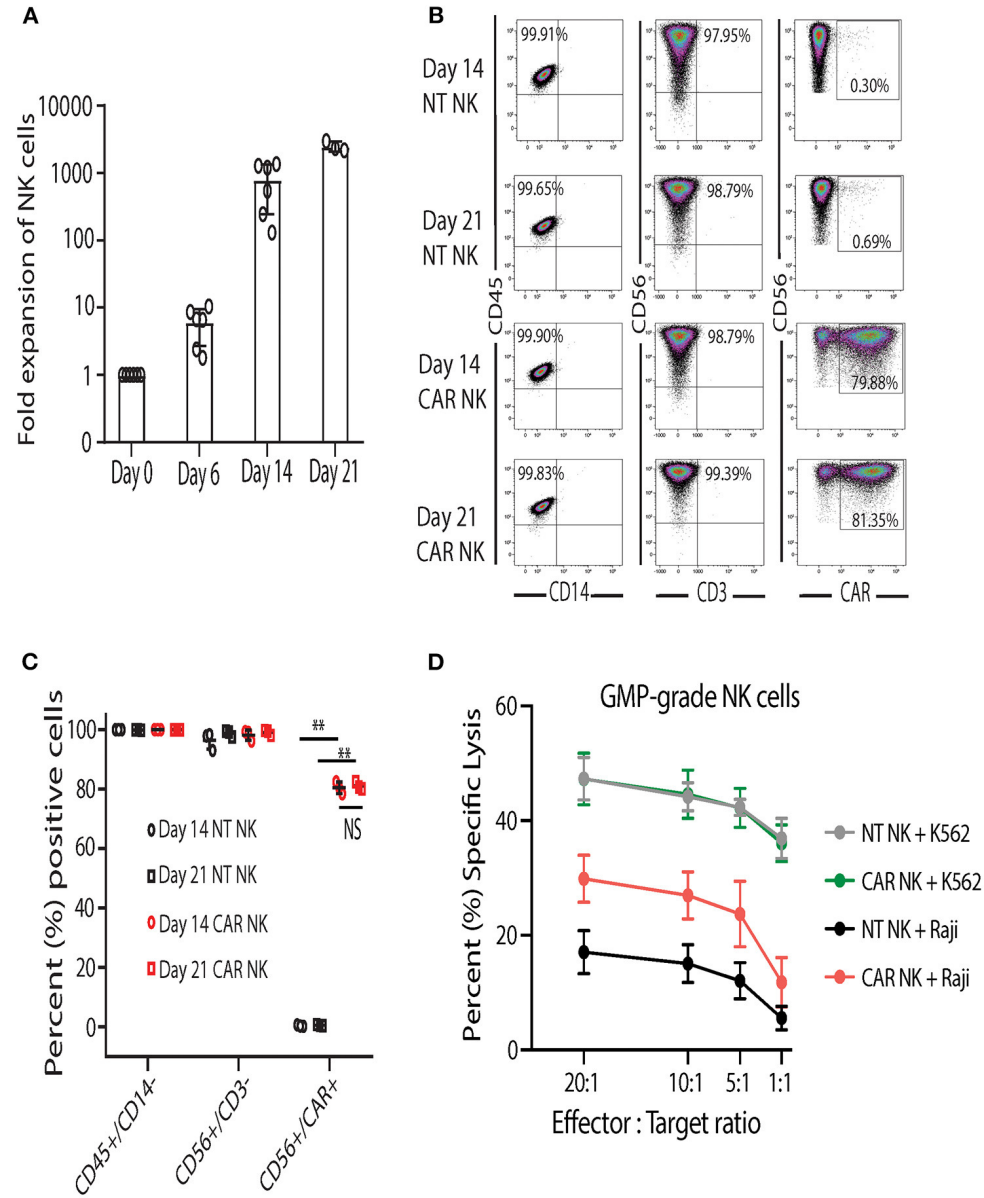
Clinical-grade NK cells co-cultured with GMP-grade uAPC display robust expansion and antitumor activity. **(A)** Fold expansion of clinical-grade CB NK at day 0 (baseline) or after co-culture with GMP-grade uAPC for 6, 14, or 21 days. Bars represent standard error of the mean (SEM) (*n* = 6 independent experiments). **(B)** Representative FACS plots showing the expression of clinical-grade NK cell lymphocytes (CD45^+^CD14^−^), GMP-grade NT (CD56^+^CD3^−^) and GMP-grade iC9/CAR.19/IL-15 (CAR; CD56^+^CD3^−^CAR^+^) NK cells at days 14 and 21 after co-culture with GMP-grade uAPC. Inset numbers are the percentages (%) positive cells within the indicated regions. **(C)** Graph summarizing the FACS data from **(B)**. Each symbol (circle or square) represents an independent experiment (*n* = 3). Statistical significance is determined as **p ≤ 0.01. Bars represent standard error of the mean (SEM). NS, not significant. **(D)** GMP-grade NT or iC9/CAR.19/IL-15 (CAR) NK cells were expanded with GMP-grade uAPC for 14 days then co-cultured for 4 h with K562 or Raji targets at different effector: target ratios and their cytotoxicity was measured by ^51^Cr release assay (*n* = 5 donors). Bars represent standard error of the mean (SEM).

After developing and establishing release criteria for GMP-grade uAPC, we validated and characterized the NK cell expansion process in the GMP laboratory. In contrast to research-grade uAPC batches, which are grown as needed and irradiated prior to use, clinical-grade uAPC batches are expanded, irradiated, cryopreserved, and thawed for use as needed. Similar to research grade CB NK cells (Figure 3A), GMP-grade CB NK cell products showed robust expansion (>1,000-fold) at days 14 and 21 after co-culture with GMP-grade uAPC (Figure 6A). Importantly, NT and iC9/CAR.19/IL-15 (CAR) NK cells expanded with GMP-grade uAPC had excellent purity, with low frequencies to zero T cells detected (0.01–0.04%) (Figures 6B,C). Low T cell contamination is a requirement to prevent GvHD in our clinical NK cell products. Further, the expanded cell populations are CD45^+^ CD32^−^, indicating no uAPC cell outgrowth (Figures 6B,C). The iC9/CAR.19/IL-15 (CAR) NK cells stimulated using GMP-grade uAPCs exerted greater cytotoxicity against Raji lymphoma cells compared to NT controls (Figure 6D), while uAPC-stimulated NT and iC9/CAR.19/IL-15 (CAR) NK cells were equally efficient at killing K562 targets (Figure 6D). Once clinical-grade uAPCs were validated in the GMP Laboratory, a master cell bank was established, and FDA approval was obtained for us in ongoing phase I/II clinical trials.

## Discussion

The relatively small numbers of NK cells found in unmanipulated cord blood or peripheral blood mononuclear cell fractions limit the therapeutic application of NK cell immunotherapy. Thus, *ex vivo* expansion of NK cells has been increasingly used to generate clinically relevant doses.

While cytokine cocktails including IL-2 have been used to activate and expand NK cells, these strategies do not yield sufficient numbers of NK cells for clinical use. This is because cellular contact is crucial to drive efficient NK cell expansion *ex vivo* (25). A number of K562-based artificial APC lines have been described and used to expand primary NK cells (26, 27), NKT cells (28), a variety of T cell subsets (29–33) and tumor-infiltrating lymphocytes (TILs) (34, 35) as well as CAR-transduced lymphocytes (25, 36–38). The transmembrane 4-1BB is currently the most commonly employed costimulatory receptor to expand NK cells along with CD80/86 and various combinations of IL-2, IL-15, and IL-21. Our approach leverages a unique SLAMF-mediated immunological sculpting of NK cells to optimize the products for clinical application. Our goal was to determine a minimal set of antigenic stimuli sufficient to trigger optimal *ex vivo* expansion of research and clinical grade NK cells within 2–3 weeks. The K562 tumor cell line was selected as stimulatory cells because they, (1) are mostly devoid of MHC determinants and thus sensitive to NK cell cytotoxicity, (2) grow in suspension culture easing the interaction with NK cells, (3) have a relatively short doubling time of 18–24 h, and (4) are easily and stably transduced by retrovirus to enforce expression of antigens as desired.

There is no known or simple substitute for “signal 1” (39) in NK cells (such as the TCR-MHC interaction in T cells). Initiation of direct physical interactions between NK cells and target cells greatly influence NK cellular responses with regard to killing or sparing of the target cells. Thus, we suggest that the “missing self” interaction mediated by KIR recognition of absence of HLA class I on uAPC is the closest surrogate to a “signal 1” response in NK cells. To induce a signal 2 (coreceptor) initiated cellular interactions, we considered using antigens from the signaling lymphocytic activating molecule (SLAM, previously also known as the CD2 superfamily) of homologous immunoglobulin receptors, which are widely expressed and play critical roles in the immune system, with a particularly important feature of intercellular interactions. We used CD48 as a surrogate “signal 2” in uAPC to initiate cell-to-cell interaction with NK cells. CD48 is a glycosylphosphatidylinositol-anchored protein (GPI-AP) found on the cell surface that participates in adhesion and activation pathways in immune cells. Despite its lack of an intracellular domain, stimulation of CD48 induces rearrangement of signaling factors in lipid rafts, Lck-kinase activity, and tyrosine phosphorylation. As an adhesion and co-stimulatory molecule, CD48 is the counter receptor for 2B4, an important activator of NK cells (40).

We also used 4-1BB ligand as a second “signal 2” to mediate NK cell proliferation and differentiation. Of note, 4-1BB was significantly upregulated in our uAPC-stimulated NK cells collected on day 15 (Figure 4A). In activated NK cells, CD137 is a cytokine-inducible costimulatory molecule, which in turn drives anti-tumor responses in NK cells by increasing cellular proliferation and IFN-γ secretion. Studies using CD137L^−/−^ knockout mice showed the importance of CD137/CD137L signaling axis in developing anti-tumor immune cells. The critical roles of CD137 in regulating NK cell-mediated anti-tumor effect is evident in CD137^−/−^ knockout mice which experience a 4-fold higher frequency of tumor metastases compared to control mice. 4-1BB ligand (4-1BBL, CD137L), forms homo-trimeric ectodomain, with a distinct three-bladed propeller conformation (41), that differs from trimers formed by other members of the tumor necrosis factor (TNF) superfamily, implying functional difference in molecular signaling (42). While it is possible to stimulate 4-1BB on NK cells with anti-4-1BB antibody, we chose 4-1BB ligand (41BBL), the physiological counter-receptor for CD137, for optimal stimulations.

Cytokine signaling is also crucial for maintenance of lymphocyte survival, proliferation, and effector efficacies. IL-2 administration *in vivo* is the only FDA-approved method for expanding immune cells in patients. Recognizing the importance of cytokine stimulations on the health of NK cells, our strategy for “signal 3” is to encapsulate mbIL-21 as part of the uAPC. For NK cells genetically engineered to express CARs, we encoded an IL-15 expression cassette for constitutive autocrine secretion (9).

While the γ_c_ cytokine receptors share similar cellular signaling components of IL-2, IL-4, IL-7, IL-9, IL-15, and IL-21 receptors in many immune cells, human NK cell-specific signaling is critically dependent on IL-21 receptor–STAT3 nexus for proliferation. STAT3 is a highly potent NK cell proliferation activator, capable of inducing NKG2D expression. The IL21R-STAT3 nexus is underscored by dampened IL-21 responses in Stat1/Stat3 double knock-out mice (43). For efficient utilization of IL-21, we enforced expression of membrane-bound IL-21 (mbIL21) on uAPC (Figure 1A) to concentrate and localize their interaction *in trans* with IL21R on NK cells. Previously, co-cultures with irradiated K562-mb15-41BBL generated a median 1,000-fold expansion of CD56^+^CD3^−^ NK cells from peripheral blood (25) after 3 weeks, compared to 2 weeks with our uAPC. This expansion is higher compared to stimulation with just soluble cytokines from the shared γ_c_ family including IL-2, IL-12, IL-15, IL-21 alone or in combinations. By comparison, we are able to expand our NK cells by up to 1,000-fold (3-log) over 14 days using uAPC for both non-transduced and CAR-transduced NK cells. CAR NK cells were highly efficient at killing CD19-positive targets such as Raji, Daudi, and primary CLL cells (44) and were superior at exerting antitumor activity in a xenograft mouse model of lymphoma compared to CAR NK cells expanded with a previously reported K562-derived APCs (C9/IL-21) expressing 4-1BBL and mbIL21 only (15), supporting the notion that a three-signal model of NK cell activation can support expansion of NK cells with greater potency. Of note, uAPC feeder cells could also efficiently expand NK cells expressing only CAR19 without the IL-15 transgene (44), further demonstrating its universal utility.

While we noted downregulation of IL-21R (Figure 4A) in NT and CAR NK cells after incubation with uAPC, the mechanism for this observation remains to be determined. Mobilization of energetics (glycolysis, oxidative phosphorylation, and fatty acid metabolism) and lipid biogenesis genes fits the metabolic profiles of proliferating NK cells (44). Importantly, the absence of exhaustion-, anergy-, or senescence-related biomarkers (Figure 4B) supports our goal of producing NK cells ready to target cancer. In addition, phenotypic profiling of NK cells by mass cytometry (Figure 5) yielded distinctive resting and activated NK cell populations, indicative of non-clonal expansion.

In summary, instead of relying solely on commercial vendors for critical biological reagents, which can result in unpredictable and deleterious supply chain disruptions, our adaptable and robust uAPC platform provides the necessary and intended NK cell stimulations in one compact package for continual clinical use. We show that phenotypically different NK cell subpopulations can be optimally expanded and molded *ex vivo* using a minimal set of universal antigenic stimulants to trigger signaling axes analogous to the three-signal model for T cell activation, in the presence or absence of CAR. Our model leverages the “missing self” interaction as signal 1, the CD48-2B4 and CD137-CD137L costimulatory interactions as signal 2, and IL-21 cytokine as signal 3 (Visual Abstract). We have moved beyond pre-clinical translational characterization of uAPC, having validated the production in the GMP setting and are currently using clinical-grade uAPC to produce NK and CAR-NK cells in MD Anderson clinical trials (21) (NCT Number: NCT03056339). As a proof of principle, we showed that uAPC can power the production of CAR NK targeting CD19 antigen (9) which is used to target B-cell malignancies. Future plans include expanding the use of CAR-NKs to target a wide range of antigens and indications including solid tumors.

## Data Availability Statement

The datasets generated in this study can be found in online repositories. The names of the repository/repositories and accession number(s) can be found below: https://www.ncbi.nlm.nih.gov/geo/query/acc.cgi?acc=GSE162716.

## Ethics Statement

The animal study was reviewed and approved by MD Anderson Cancer Center.

## Author Contributions

EL, SAn, RC, ES, and KR conceived and designed the experiments. EL, SAn, ES, and KR designed the transfer constructs. EL, IK, ES, and KR developed the uAPC expansion SOPs. EL, LK, RB, MK, LL, YT, MD, EE, NU, and AN performed the experiments. EL, SAn, LK, RB, MD, RY, YL, HS, FR, PB, IK, VM, SAc, MS, PL, KC, RC, ES, and KR analyzed the data. EL and SAn contributed reagents/materials/analysis tools. EL, SAn, LM-F, and KR wrote the manuscript. All authors contributed to the article and approved the submitted version.

## Conflict of Interest

KR, ES, RC, EL, SAn, RB, MD, PB, and The University of Texas MD Anderson Cancer Center (MDACC) have an institutional financial conflict of interest with Takeda Pharmaceutical for the licensing of the technology related to CAR-NK cell research reported here. MD Anderson has implemented an Institutional Conflict of Interest Management and Monitoring Plan to manage and monitor the conflict of interest with respect to MDACC's conduct of any other ongoing or future research related to this relationship. KR, ES, RB, EL, SAn and The University of Texas MD Anderson Cancer Center has an institutional financial conflict of interest with Affimed GmbH. Because MD Anderson is committed to the protection of human subjects and the effective management of its financial conflicts of interest in relation to its research activities, MD Anderson is implementing an Institutional Conflict of Interest Management and Monitoring Plan to manage and monitor the conflict of interest with respect to MD Anderson's conduct of any other ongoing or future research related to this relationship. KR participates on Scientific Advisory Board for GemoAb, AvengeBio, Kiadis, GSK and Bayer. The remaining authors declare that the research was conducted in the absence of any commercial or financial relationships that could be construed as a potential conflict of interest.

## Abbreviations

B-ALL: B cell acute lymphoblastic leukemia
CAR-T: chimeric antigen receptor T cell
DLBCL: Diffuse large B-cell lymphoma
uAPC: universal antigen presenting cell.
