# Supplementary material for: GMP-Compliant Universal Antigen Presenting Cells (uAPC) Promote the Metabolic Fitness and Antitumor Activity of Armored Cord Blood CAR-NK Cells
Source: Front Immunol. 2021 Feb 26;12:626098. doi: 10.3389/fimmu.2021.626098 (PMC7952299; doi:10.3389/fimmu.2021.626098)
Supplement: Supplementary file 1 [file Data_Sheet_1.pdf]

Supplementary Materials

Supplementary Figures

Supplementary Figure 1

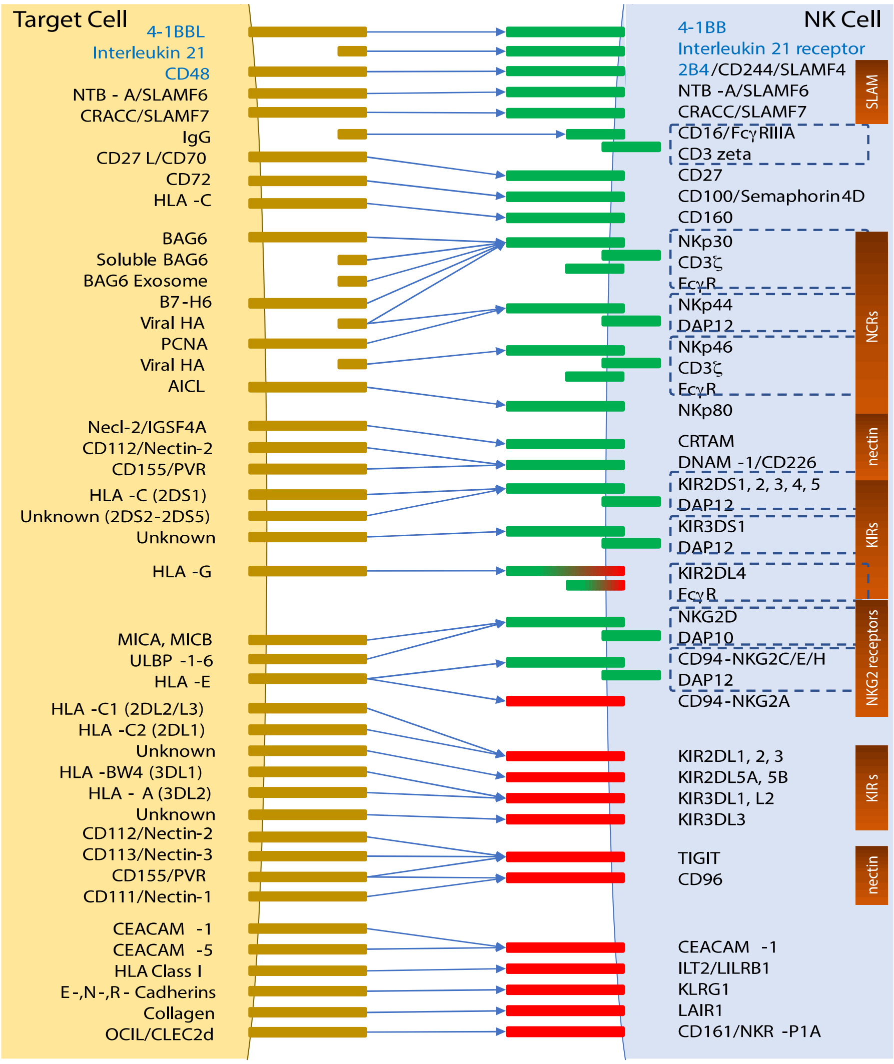

Supplementary Figure 1. Human NK cell and target cell interactions. Human NK cells (right) interact with target cells (left) via a myriad of receptors, including killer immunoglobulin receptors (KIRs), natural cytotoxicity receptors (NCRs), NKG2 family of receptors, nectin binding receptors (TIGIT, CD96, DNAM, CRTAM), signaling lymphocytic activation molecule (SLAM) family receptors (2B4, CD48, NTB-A, CRACC) and others. Receptors on NK cells can be activating (green) or inhibitory (red). We generated a uAPC cell line to target three critical signaling pathways (blue) on NK cells namely 2B4 (CD244), 4-1BB (CD137), and IL-21 receptor.

Supplementary Figure 2

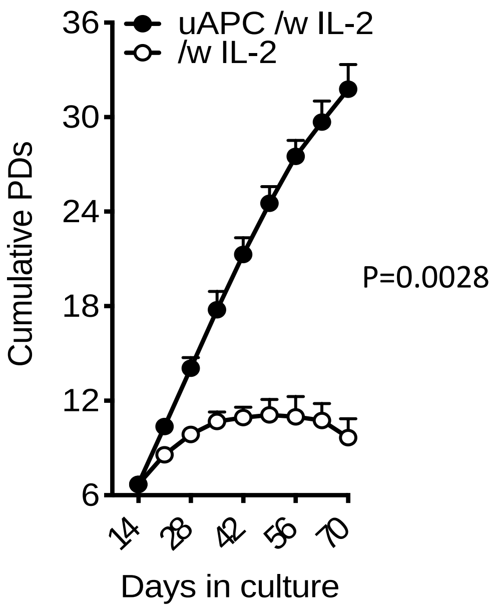

Supplementary Figure 2. Fold expansion of NK cells cultured with uAPC + IL-2 vs IL-2 alone in the absence of feeder cells. iC9/CAR.19/IL-15 (CAR) NK cells were cultured with uAPC + IL-2 for one week. The cultures were then split into two and NK cells were expanded either with IL-2 (100 U/ml) + weekly uAPC feeder cells or with IL-2 alone (100 U/ml) without uAPC feeder cells. The population doubling (PD) was calculated using the following formula: PD=(A/B)/log2, where A is the number of harvested cells and B the number of plated cells from each subculture. The accumulated PD per passage was greater in NK cells expanded with uAPC + IL2 compared to NK cells expanded with IL-2 alone; n=3 independent experiments. The statistical significance was determined using two-way ANOVA, P=0.0028.

Supplementary Figure 3

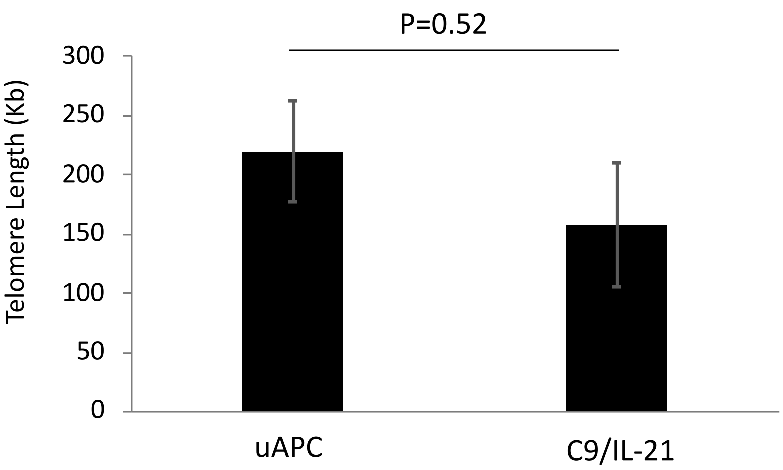

Supplementary Figure 3. Telomere lengths of NK cells expanded with C9/IL-21 + IL-2 vs uAPC + IL-2. iC9/CAR.19/IL-15 (CAR) NK cells were co-cultured in parallel using either uAPC (left) or C9/IL-21 (right) feeder cells. The average total telomere length per cell from each group was determined by quantitative polymerase chain reaction (qPCR) and there is no statistically significant difference between CAR NK cells co-cultured with uAPC vs the previously characterized C9/IL-21 (n=6).

Supplementary Figure 4

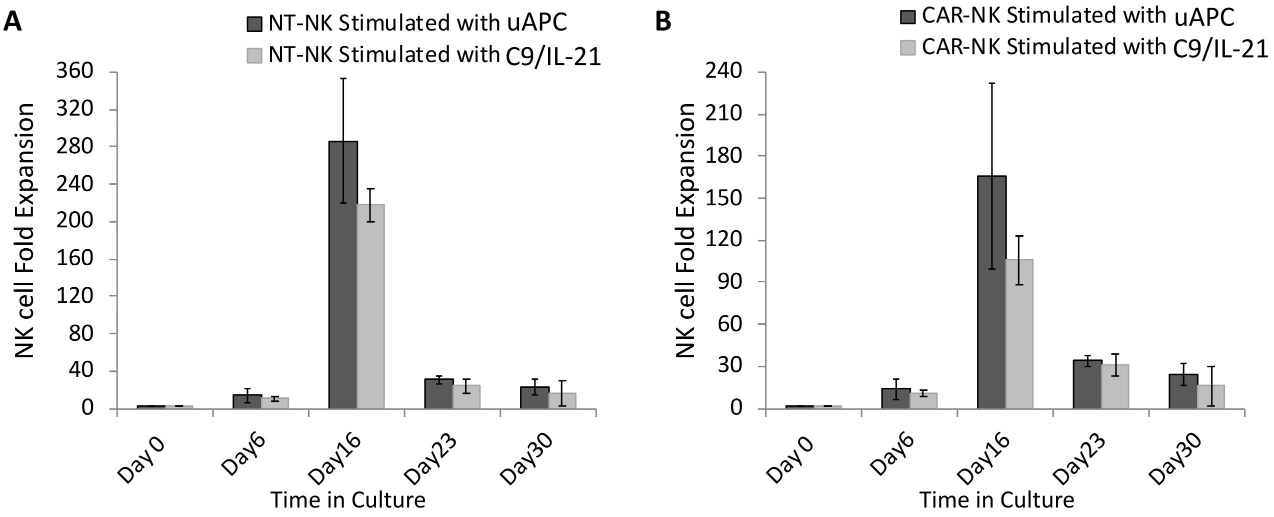

Supplementary Figure 4. Fold Expansion of NT or CAR-NK cells cultured with uAPC + IL-2 vs. C9/IL-21 + IL-2. Fold expansion of NT (**A**) or iC9/CAR.19/IL-15 (CAR) (**B**) NK cells cultured with uAPC + IL-2 (100 U/ml) (dark grey bars) vs C9/IL-21 + IL-2 (100 U/ml) (light grey bars) for 6, 16, 23 or 30 days. There was no significant difference in the fold expansion between NT-NK cells and CAR-NK cultured with uAPC + IL-2 vs C9/IL-21 + IL-2 (n=3 independent experiments).

Supplementary Figure 5

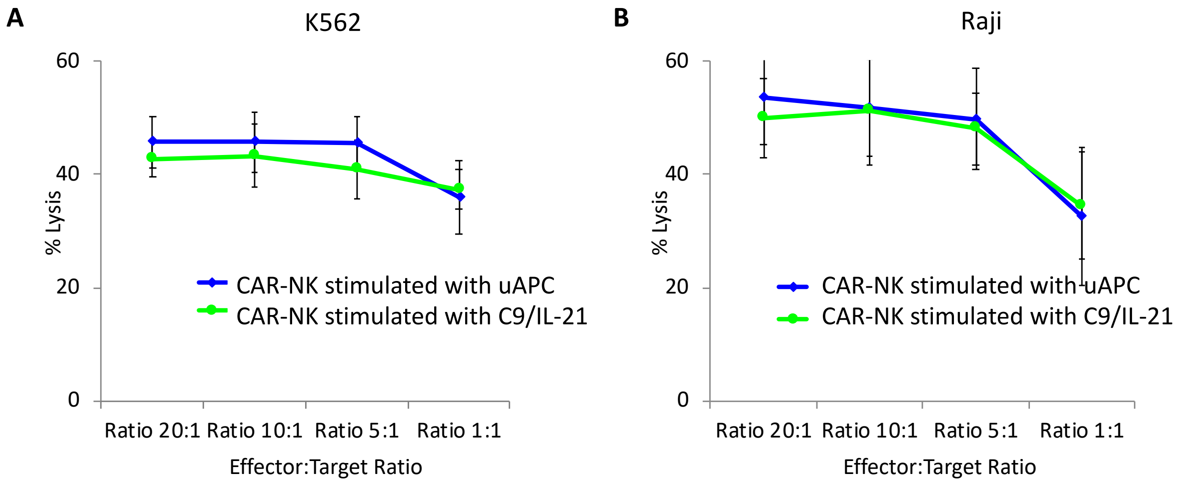

Supplementary Figure 5. Antitumor activity of iC9/CAR.19/IL-15 (CAR) NK cells expanded with uAPC or C9/IL-21. iC9/CAR.19/IL-15 (CAR) NK cells were expanded with uAPC (blue line) or C9/IL-21 (green line) and their cytotoxicity against K562 (**A**) or Raji (**B**) targets was measured by a four hour ^{51}Cr release assay (n=3 independent experiments). Bars represent standard error of the mean (SEM).

Supplementary Figure 6

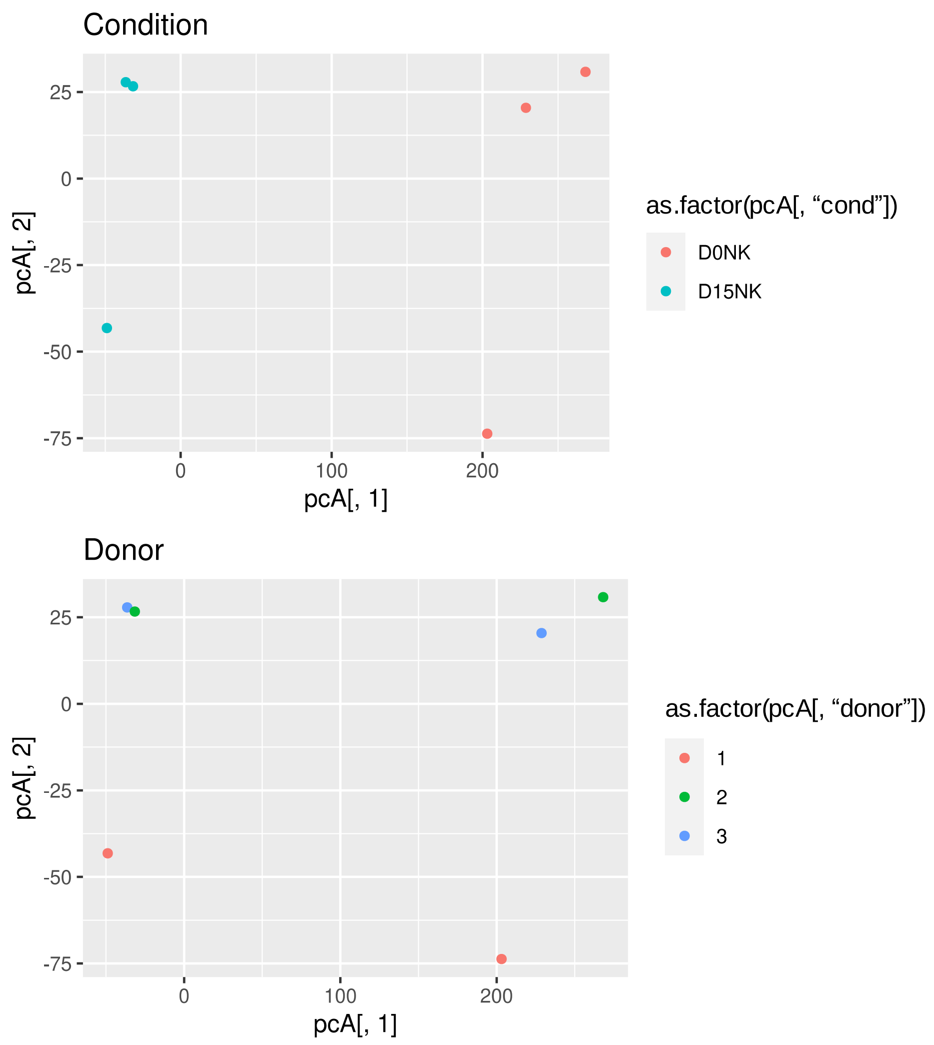

Supplementary Figure 6. Principal component analysis (PCA) to differentiate NK cells before and after uAPC co-incubation. PCA was used in initial data analysis and predictive modeling to characterize the genetic distances as depicted in 2-dimensional projections. PCA revealed high genetic variance-covariance which contribute to the low relatedness between NK cell populations in the multivariate dataset, highlighting the non-clonal repertoire of studied donors. D0NK = day 0 unstimulated, freshly isolated NK cells. D15NK = day 15 post uAPC stimulated NK cells.

Supplementary Tables

Supplementary Table 1. List of antibodies used in Flow cytometry

Antigen	Antibody clone	Fluorophore	Source
CD48	BJ40	FITC	Biolegend
IL-21	3A3-N2.1	Brilliant Violet 421	BD Biosciences
CD37L	5F4	PE	Biolegend
CD32	FLI8.26	FITC	BD Biosciences
CD14	MφP9	V450	BD Biosciences
CD45	2D1	V500-C	BD Biosciences
CD3	HIT3a	APC/Cy7	Biolegend
CD56	HCD56	Brilliant Violet 605	Biolegend
CAR	Goat F(ab')2 anti-Human IgG (H+L)	Alexa Fluor 647	Jackson Laboratories
CD107a	H4A3	Brilliant Violet 785	Biolegend
TNF-α	MAb11	Alexa Fluor 700	eBioscience
IFN-γ	B27	V450	BD Biosciences
(live/dead)			Thermo Fisher

Supplementary Table 2

List of antibodies used for mass cytometry to characterize the NK cell repertoire

Tag	Antibody	Clone	Source
142Nd	EOMES	WD1928	Thermo Fisher
143Nd	ZAP70	1E7.2	Biolegend
144Nd	Siglec 7	194211	R&D
145Nd	CD62L	DREG-56	BD Bioscience
146Nd	CD8a	RPAT8	BD Bioscience
147Sm	NKG2C	134591	R&D
149Sm	CD25	2A3	BD Bioscience
154Sm	TIGIT	MBSA43	Thermo Fisher
155Gd	CD27	M-T271	BD Bioscience
156Gd	KLRG1	13F12F2	Thermo Fisher
158Gd	CD94	DX22	Biolegend
159Tb	NKP30	Z25	Fluidgm
161Dy	T-BET	4B10	Biolegend
162Dy	NKP46	BAB281	Fluidgm
166Er	NKG2D	BAT221	Miltenyi
167Er	2B4	C1.7	Thermo Fisher
169Tm	NKG2A	Z199	Fluidgm
171Yb	DNAM	Dx11	BD Bioscience
172Yb	PRFN	B-D48	Abcam
173Yb	GRANZYME B	GB11	BD Bioscience
174Yb	GRANZYME A	CB9	Biolegend
176Yb	CD56	NCAM16.2	BD Bioscience
209Bi	CD16	3G8	Fluidgm
115In	CD57	HCD57	Biolegend
194Pt	CD7	M-T701	BD Bioscience
145Nd	2DS1	1127B	R&D
149Sm	2DS4	MAB1847	R&D
172Yb	3DL1	DX9	Biolegend
173Yb	DS1	REA1010	Miltenyi
164Dy	c-kit	104D2	Biolegend
171Yb	CCR7	G043H7	Biolegend
148Nd	CD158b	DX27	Biolegend
169Tm	KIR2DL1	HP-DM1	Biolegend
156Gd	KIR2DL3	FAB2014F	R&D
172Yb	KIR3DL1	DX9	Biolegend
174Yb	Syk	MAB7166	R&D
198Pt	Live/dead		Fluidgm

Supplementary Table 3

uAPC STR profile (cell line fingerprint) comparisons with DSMZ and ATCC database entries

Marker	uAPC	DSMZ/ATCC
AMEL	X	X
CSF1PO	9,10	9,10
D13S317	8	8
D16S539	11,12	11,12
D18S51	15,16	15,16
D21S11	29,31	29,31
D3S1358	16	16
D5S818	11,12,13	11,12,13
D7S820	9,11	9,11
D8S1179	12	12
FGA	21,24	21,24
TH01	9.3	9.3
TPOX	8,9	8,9
vWA	16	16

STR (Short Tandem Repeat)
DSMZ (German Collection of Microorganisms and Cell Cultures GmbH)
ATCC (American Type Culture Collection)
